# Neurodiversity-Affirming Applied Behavior Analysis

**DOI:** 10.1007/s40617-024-00918-0

**Published:** 2024-03-25

**Authors:** Lauren Lestremau Allen, Leanna S. Mellon, Noor Syed, Joy F. Johnson, Armando J. Bernal

**Affiliations:** 1 SUNY Empire State University (Applied Behavior Analysis), Saratoga Springs, NY USA; 2 Center for Autism Advocacy: Research, Education, & Supports (CAARES), Saratoga Springs, NY USA; 3 Ivymount School, Rockville, MD USA; 4 State University of New York at New Paltz (Teaching & Learning), New Paltz, NY USA; 5 Anderson Center International, Anderson Center for Autism, Staatsburg, NY USA; 6 Endicott College, Beverly, MA USA; 7 Spectrum Support, Baltimore, MD USA; 8 Autism International Consulting, PLLC, Spring, TX USA

**Keywords:** Neurodiversity-affirming, Dignity, Self-determination, Assent, Social validity, Ableism

## Abstract

Individuals within the Autistic and Neurodivergent communities have shared numerous concerns about applied behavior analysis (ABA). These criticisms often relate to the ableism reflected within current practices, which have impeded the dignity and autonomy of many individuals with disabilities served through ABA. Both within the field and outside of the field, there is a growing acknowledgment of the need to listen, reflect, and reconsider approaches to service delivery, which can ultimately benefit service recipients well beyond the Autistic or Neurodivergent communities. ABA is committed to being responsive to consumers, even when the social validity data are unfavorable, and the path forward is unclear. This article will provide an overview of historical and current perspectives regarding disability rights, the Autistic and Neurodiversity advocacy movements, and disability as a form of diversity. Calls to action will be presented with accompanying neurodiversity-affirming actions for behavior analytic practitioners. These calls to action are informed by feedback from the Autistic and Neurodivergent communities as well as other interested parties and are related to (1) client identity and language; (2) dignity, self-determination, choice, and assent; and (3) social validity, which may be acted on through compassionate and affirming approaches.

## Introduction

Many p﻿rofessionals within the field of applied behavior analysis (ABA) have focused on service to Autistic individuals and individuals with other developmental disabilities (Behavior Analyst Certification Board [BACB] n.d.). Over 81% of Board Certified Behavior Analysts® (BCBA) provide services to Autistic clients (BACB, n.d.). The use of ABA is considered evidence-based practice with Autistic individuals (Hume et al., 2021; National Autism Center, 2015; Odom et al., 2010; Wong et al., 2015), with randomized controlled trial studies demonstrating its efficacy (e.g., Dixon et al., 2021). For this reason, ABA is a commonly recommended intervention, particularly for caregivers of young, newly diagnosed Autistic children (Mulé et al., 2022). The science of behavior analysis is not limited in application to a specific population (Heward et al., 2022). However, the predominance of ABA as an approach used with Autistic individuals has been shaped by decades of advocacy and legislation that has resulted in funding through insurance mandates (Trump & Ayres, 2020). This history has resulted in the misconception that ABA is a “treatment” (Sigafoos & Schlosser, 2008) intended to “cure” autism.

Despite ABA’s widespread use with Autistic and Neurodivergent individuals, many concerns have been leveled against the field from those in these communities as well as others (Autistic Self Advocacy Network [ASAN], 2024c; Cernius, 2022; Devita-Raeburn, 2016; Lynch, 2019; Neuroclastic, 2020; Ram, 2020; Sandoval-Norton & Shkedy, 2019; Shyman, 2016; Stop ABA, Support Autistics, n.d.; Stout, n.d.; Wilkenfeld & McCarthy, 2020). The criticisms primarily relate to the harm caused by ABA (e.g., Anderson, 2022; National Council on Independent Living [NCIL], 2021; Ne'eman, 2021). Many critics would suggest that client progress or perceived gains were made at the expense of client well-being and that the “ends do not justify the means” (Shkedy et al., 2021, p. 127). Examples of these “means” may include behavior analysts attempting to (1) “cure” autism, a condition that is not pathological (Ne’eman, 2010, curing autism section); (2) “normalize” Autistic individuals (Ne’eman, 2010, curing autism section; Shyman, 2016, p. 371); and (3) render Autistic individuals as indistinguishable from the neuromajority (term used in place of “neurotypical”; Anderson, 2022; Veneziano & Shea, 2023).

Behavior analysts often serve Autistic and Neurodivergent individuals without graduate coursework related to disability rights, ableism, and neurodiversity, given the coursework requirements outlined by the BACB (2022b). The former behavior analytic ethical code, in place before January 2022, mentioned dignity and respect only in the context of participation in research (BACB, 2014). This earlier version of the code also did not mention social validity (BACB, 2014). Although client rights are expected to be prioritized within service delivery, promoting these rights may be constrained if there is not clarity regarding *who* the client is and what their rights *are*. The former code outlined the client’s “right to effective treatment” (BACB, 2014, p. 8) but neglected to address that “effectiveness” is not the only metric that matters. Further, “effectiveness” determined by whom? Although consideration of client rights should be at the forefront of all service delivery, promotion of client rights may be constrained if there is not clarity regarding who the client is and what their rights are.

Improvements were made in the recently adopted ethical code (BACB, 2020), including the addition of the core principle to “treat others with compassion, dignity, and respect” (p. 4) and the language regarding respect and client self-determination. However, it is not yet clear whether these changes are sufficiently substantive and whether unintentional harm may have been caused due to their historical absence. It is important to note that criticisms regarding ableist practices are not isolated to ABA; for example, concerns exist regarding special and general education (Timberlake, 2020), occupational therapy (Hammell, 2022; Yao et al., 2022), and speech/language pathology (DeThorne & Gerlach-Houck, 2022). However, along with other fields, “we must be ready to genuinely apologize and commit to doing better. Each event in our personal and professional history is a learning opportunity” (Kirby et al., 2022, p. 151).

### Purpose

The co-authors seek to demonstrate how neurodiversity and disability affirming services can be provided utilizing applied behavior analysis. An overview of historical and current perspectives regarding neurodivergence and disability including disability rights, advocacy movements, and the relationship between disability and diversity will be offered. Calls to action, informed by criticisms from Autistic and Neurodivergent communities and other interested parties, will be outlined. These calls to action focus on the issues of client identity and language; self-determination, choice, and assent; and social validity and may be considered within individual practice as well as the systems and structures in which individual practitioners and researchers operate.

### Commitment to Representation

This article was developed in collaboration between individuals of different neurotypes, disability statuses, ethnic/racial backgrounds, genders, and cultures.^1^ The authors acknowledge that the breadth of perspectives and experiences of those within the Autistic and Neurodivergent communities cannot be reflected in one article. Yet, the co-authors strived to offer information from a variety of sources. This article was informed by the published, peer-reviewed literature and first-person experiences and media created by Autistic and Neurodivergent individuals (e.g., social media, websites, blogs). Conversations the authors have shared with Autistic and Neurodivergent individuals and the Autistic and Neurodivergent authors’ lived experiences also informed this article. Identity-first language is used throughout this article out of respect for the current majority preference by Autistic individuals and “Autistic” is capitalized to reflect the culture of this community (Pittsburgh Center for Autistic Advocacy, 2023).

### Ableism and Implicit Bias

The throughline of criticisms regarding ABA center on ableism within applications of our science. Matsuda et al. noted in 2020 that our field had yet to offer technical definitions for terms such as racism; the same is true for ableism. Ableism reflects beliefs that nondisabled individuals are the “standard” or are “normal” (Stop Ableism, 2023, para. 4), and, therefore, disabled individuals require fixing (Center for Disability Rights, n.d.). Ableism is a

belief system analogous to racism, sexism, or ageism that sees persons with disabilities as less worthy of respect and consideration, less able to contribute and participate, or of less inherent value than others. Ableism may be conscious or unconscious and embedded in institutions, systems, or the broader culture of a society. It can limit the opportunities of persons with disabilities and reduce their inclusion in the life of their communities (Law Commission of Ontario, 2012, p. 2).

Implicit bias contributes to the development and maintenance of ableism (Huang et al., 2023). Implicit bias has long been thought to be outside the purview of ABA (Jaramillo & Nohelty, 2022). Extensions can be made from the limited but growing body of behavior analytic literature related to racism (Machado & Lugo, 2022; Matsuda et al., 2020; Jaramillo & Nohelty, 2022). For example, implicit biases have been operationalized a “set of behaviors that can be changed” (Jaramillo & Nohelty, 2022, p. 1181) and as “something people do rather than something that people possess” (De Houwer, 2019, p. 836). Ableism, therefore, reflects not only private but also observable behaviors, and these “biased behaviors” can be measured (Matsuda et al., 2020, p. 337). Mathur and Rodriguez (2022) define implicit bias as when one’s “private behaviors . . . (e.g., a diagnostician saying to themselves, ‘I diagnose all children equitably’) are inconsistent with their overt behavior (e.g., diagnosing Black children with ASD at a later age or after more follow up visits, than white children. . . .”; p. 1024). For a discussion of how biased behaviors may develop within a behavior analytic or a functional-contextual framework, interested readers are directed to Matsuda et al. (2020). Some examples of ableism reflected in behavior analytic applications include:

- Targeting goals based on the caregiver’s, behavior analyst’s, or society’s perceptions of “typical” functioning, as opposed to the client’s quality of life;
- Classifying the response of “no” as noncompliance for an Autistic client and as self-advocacy or assent withdrawal by a neuromajority individual; and
- Intervening to reduce hand flapping or spinning by Autistic individuals while accepting the foot tapping by neuromajority people as innocuous.

As is demonstrated in these examples, the *Ethics Code for Behavior Analysts* notes that behavior analysts should “evaluate their own biases and ability to address the needs of individuals with diverse needs/backgrounds . . .” because these biases may impede professional effectiveness (BACB, 2020, p. 9).

### History and State of Disability Rights and Advocacy

Although the *Ethics Code for Behavior Analysts* (BACB, 2020) *assumes* practitioners are aware of biases, Arango and Lustig (2023) posit that behavior analysts are often either unaware of or unintentionally downplay the importance of differences across cultural groups. For this reason, it is important to remain humble, meaning to acknowledge that one cannot be fully competent in an identity that is not their own. One can be humble by engaging in self-reflection (Wright, 2019) via seeking an understanding of others’ experiences. Doing so demonstrates diligence in addressing areas outside of one’s knowledge (Arango & Lustig, 2023). An overview of critical areas within disability rights and advocacy will be offered in pursuit of building knowledge regarding the lived experiences of others, particularly as it relates to disability.

#### Medical and Social Models of Disability

Increases in state-mandated health insurance coverage for autism-related health care, including behavioral health care, were associated with substantial growth in the demand for and the supply of behavior analysts (McBain et al., 2020). From 2003 to 2017, there was an approximate 12x increase compared to a 3x increase in BCBAs in states with insurance mandates versus states without, respectively (McBain et al., 2020). For this reason, most behavior analytic services in the United States^2^ have aligned to the medical model of disability to meet insurance plan requirements. The medical model focuses on a deficit framework, in which individuals are viewed as needing to normalize their behavior even when behaviors are not dangerous (Shakespeare, 2006). Societal norms are set as the expectation, and disabled individuals must adapt to fit the expectations of the nondisabled community (Shyman, 2016). Behaviors commonly accepted in society, or in particular, by the neuromajority, may become long-term goals (e.g., Rosenzweig & Prizant, 2022) without consideration of whether engaging in these behaviors would improve the lives of disabled individuals.

By contrast, the social model of disability focuses on the need for society or the *environment *to adapt and accommodate rather than the individual needing to change (Gallagher et al., 2014). The social model asserts that disability results from an environmental failure to support one’s needs rather than the brain’s diversity (den Houting, 2019; Graber & Graber, 2023; Jaarsma & Welin, 2012). Rather than assuming behaviors must be altered to fit societal norms, the social model posits that the environment should ideally be adapted to support how each individual best navigates the world to optimize their quality of life. This is akin to the Universal Design approach, in which environments and resources are created multimodally, including stairs and a ramp to access physical spaces or online textbooks with text-to-speech capability (Rose, 2000). For example, repetitive behaviors are sometimes targeted for reduction to enhance an individual’s access to more settings. Through a social model lens, the inclusivity of the individual’s environment may be targeted by teaching those within the environment to accept innocuous repetitive behaviors as a variation of behavior and educating *others* in inclusive practices so that individuals are able to self-soothe in ways they choose. This approach, therefore, allows for quality of life to be advanced through *both* increasingly inclusive environments and skill development that contributes to an individual’s autonomy (e.g., self-advocacy, communication; Dwyer, 2022).

#### History in the United States

Although the term *ableism* only expanded outside of disability rights circles more recently (Diedrich, 2023), discrimination against disabled individuals and groups dates back, some scholars note, to the early Romans and Greeks (Penrose, 2015). In the United States, it was not uncommon in the 19th and early 20th centuries for disabled individuals to be considered unfit for society and unable to contribute meaningfully (Anti-Defamation League Education, 2017; Nielsen, 2012). As a result of disability rights advocacy, the Rehabilitation Act of 1973, which prohibits discrimination against disabled people in federal matters such as employment, was passed. In 1975, the U.S. Congress enacted the Education for All Handicapped Children Act (EHA, 1975) to support states in protecting the rights and meeting the needs of children and families with disabilities. Before this time, many children were denied educational access. In 1990, EHA became the Individuals with Disabilities Education Act (IDEA, 1990, 2004), which was reauthorized in 2004. IDEA’s function is to ensure that all children have access to a free and appropriate public education or FAPE in the least restrictive environment, that educators and parents have the necessary tools for educational progress, and to assist in early intervention (U.S. Department of Education, n.d.). Finally, the Americans with Disabilities Act, initially enacted in 1990 and later revised in 2008, prohibits discrimination against disabled individuals in employment, transportation, public accommodations, and access to government services. However, it is important to note that though these laws are currently in place, ableist practices within society persist (Ne’eman, 2021).

#### Neurodiversity Movement and Autistic Advocacy

“Neurodiversity” is a term that describes the many ways human brains process information, whereas the neurodiversity *movement* reflects the social justice work aimed at pursuing rights for neurodivergent individuals (ASAN, 2024d, neurodiversity section). Neurodivergent individuals have differences in neurocognitive functioning as compared to the neuromajority, and include individuals with conditions including, but not limited to, autism, attention deficit hyperactivity disorder, anxiety, depression, dyslexia, learning disabilities, and intellectual disability (Hughes, 2016). The neurodiversity movement reflects an effort by Autistic advocates to promote disability as a positive identity (Orsini, 2009). The movement calls attention to the struggle for civil rights that many Neurodivergent individuals have faced, emphasizing that disability is diversity (University of California, Santa Barbara, n.d.). Many in the advocacy movement are calling for a shift from normative agendas in practice and research (Leadbitter et al., 2021; Ne’eman, 2021), although it is important to note that there is some dissent across groups of caregivers and self-advocates, for example (Bertilsdotter Rosqvist et al., 2015; Ne'eman & Bascom, 2020). Rather than pursuing a neuromajority model of behavior, these advocates emphasize the need for environmental modifications driven by Autistic priorities, asking “What are the barriers to accessibility and inclusion?” (Enright, 2021, third sentence).

### Validating the Critiques and Moving Forward

Despite ABA’s status as an evidence-based practice due to its effects on various client outcomes, the unintended effects of behavior analytic services are poorly understood (McGill & Robinson, 2020). Adult Autistic respondents, in a recent qualitative evaluation, reported some positive long-term effects of their prior experience of ABA (e.g., communication also described by one respondent as the “gift of speech,” functional skills, emotional identification, personal space), yet emphasized the secondary, long-term, harmful effects on bodily autonomy, mental health as a result of “dehumanization,” and academic outcomes due to their perceptions that ABA took precedence over academics (Anderson, 2022, p. 9). Other researchers have warned of this group's possible increased rates of posttraumatic stress disorder due to ABA (Kupferstein, 2018; Sandoval-Norton & Shkedy, 2019). Although some in the field have refuted these claims, criticized the methodology of the articles, or categorized responsiveness to criticisms of ABA as unethical, examples of failures to prioritize dignity span decades.

*Screams, Slaps, & Love* (Grant, 1965) was a *Life* magazine article that brought Lovaas’s work with Autistic children to the mainstream. With mentions of slaps, “scoldings and stern shakings” (Grant, 1965, Punishment for Pamela section) and contingent electric skin shock (CESS; Blenkush & O’Neil, 2020), it would be difficult to argue that the science of behavior analysis has been employed compassionately in all instances. For example, use of unconditioned punishers to reduce behavior fails to meet “the standards of least restrictive alternative of best practice” (Cooper et al., 2020, p. 348). The committed and persistent activism by disability self-advocates to #stoptheshock or ban the use of CESS (e.g., ADAPT, 2023b; ASAN, 2024a; Neuroclastic, n.d.) and the more recent statements against CESS by behavior analytic professional organizations (Association for Behavior Analysis International, 2022; Massachusetts Association for Behavior Analysis, 2021; Vanderbilt Kennedy Center Treatment & Research Institute for Autism Spectrum Disorders, 2022) highlight the potential for harm from misapplication of behavior analytic principles. McGill and Robinson (2020) urge behavior analysts to respect and honor Autistic expertise by “experience” (conclusion section).

These co-authors seek to echo voices highlighting our field’s long-standing commitment to be responsive to consumers (Baer et al., 1968; Schwartz & Baer, 1991; Schwartz & Kelly, 2021; Wolf, 1978), even when the consumer feedback is critical, and the path forward is unclear. In *They Have a Voice; Are We Listening?*, Veneziano and Shea (2023) note that it would be a “mistake” to fail to seek feedback from former recipients of behavior analytic services and that failing to do so would be “an intentional form of ableism, dismissive of the criticisms of former service recipients based on their disability” (p. 128). The need to approach criticisms curiously, humbly, compassionately, and in a manner aligned with conflict resolution instead of rebutting or dismissing concerns is paramount (Miller, 2021, p. 313). Many within our field have noted the value in and of seeking feedback, which Schwartz and Kelly (2021) suggest should be viewed as an “authentic measure of social validity” (p. 162). When this feedback is used to occasion self-reflection, it serves to improve not only behavior analytic practice but also perceptions by others (Rajaraman et al., 2022), both of which are essential if we want our science to contribute to societal change and improvements to quality of life that our field hopes to promote. Categorizing disability as a form of diversity and through a lens that values cultural differences may support these efforts.

### Disability as Diversity

Failing to identify variables related to diversity within practice serves as a barrier to providing supports that lead to socially significant change (Beaulieu et al., 2018; Jimenez-Gomez & Beaulieu, 2022; Miller et al., 2019). Diversity without consideration of disability is incomplete. Many advocates note the “disability culture” that is formed through the community’s shared experiences and resilience, with members desiring to be “known authentically, treated equally and understood by others” (Frost et al., 2019, p. 273). The neurodiversity community is viewed as possessing a “minority group identity,” contributing to and benefitting from community support and advocacy (e.g., Kapp et al., 2013, p. 2). The disability community’s efforts to obtain civil rights are similar to others’ efforts related to rights for individuals regardless of race, ethnicity, gender, and sexuality (Armstrong, 2017), emphasizing that “disability rights are civil rights” (ADAPT, 2023a, tagline). For example, disability rights advocacy has been documented since the 1800s (Longmore, 1987) and since that time has resulted in one of the earliest documented sit-ins in United States modern history (e.g., 1935 in New York City for work access relief) and the “longest nonviolent occupation of a federal building in U.S. history” (i.e., “504 Sit-In”; Longmore & Goldberger, 2000; Lu, 2021). Disability rights advocates seek to advance the understanding that disability is a “natural and valuable” form of diversity (Leadbitter et al., 2021, p. 2). Rakshit (2022) reflects that “the neurodiversity movement is gradually changing the focus of autism-related healthcare from ‘cure’ to ‘care’” (para. 1).

Both within (Graber & Graber, 2023; Juarez, 2022; Veneziano & Shea, 2023) and outside the field of ABA (DeThorne & Gerlach-Houck, 2022; Hammell, 2022), there is a growing acknowledgment of the need to listen, reflect, and reconsider our approach to service delivery with disabled individuals. Given the neurodivergent or disabled cultural identity for many individuals served within behavior analysis, emphasis on cultural humility (Wright, 2019) and cultural responsiveness (Beaulieu & Jimenez-Gomez, 2022) may serve as a framework through which this shift may occur. Cultural humility acknowledges the difficulty in becoming “competent” in another’s culture and encourages practitioners to commit to lifelong learning through self-reflection and self-critique (Wright, 2019, p. 805). Wright (2019) suggests that self-management (Cooper et al., 2020) may be the means through which behavior analysts engage in self-reflection. Cultural responsiveness extends reflection of self to practice through ongoing collaboration with clients and interested parties to ensure that assessment, goals, and interventions reflect the relevant cultural variables of those individuals or groups (Kristiansen, 2023). Behavior analytic literature on responsive practices suggests that self-awareness, meaning discriminating between and tacting contingencies for oneself and others (Dymond & Barnes, 1997; Skinner, 1953, 1974), may be a prerequisite skill for responsive practices (Fong et al., 2016; Rohrer et al., 2021). Through cultural humility and responsiveness, biases may be identified and addressed (Wright, 2019) and understanding may be increased (Arango & Lustig, 2023; Beaulieu & Jimenez-Gomez, 2022), ensuring the priorities and values of Neurodivergent and disabled clients and their communities are reflected in the behavior analytic care they receive. Acknowledging the significance, the *Sixth Edition BCBA Test Content Outline* (BACB, 2022a) includes revisions to incorporate cultural humility and cultural responsiveness, and BCBA coursework requirements that go into effect by 2027 require inclusion of diversity, equity, and inclusion in content related to ethics, assessment, intervention, and organizational behavior management.

### Compassionate and Neurodiversity-Affirming Approaches

The need to address ableism within ABA relies on approaches that are compassionate (Taylor et al., 2019) and affirm disability as an identity. Although much of the compassionate care literature in behavior analysis has focused on collaboration with caregivers (Rohrer et al., 2021; Taylor et al., 2019), application of affirming and compassionate approaches within client interactions and direct service delivery is critical. Across behavior analytic and person-centered therapy literature, compassionate care (Taylor et al., 2019) and unconditional positive regard (Raskin, 2001) emphasize the role of celebrating or prizing the client, respectively, within a therapeutic relationship. The Autism Society of America (2023) identified the importance of unconditional positive regard within ABA. Yet, although respondents in Taylor et al. (2019) rated behavior analysts high for celebrating or appreciating clients, this reinforcement was provided *contingently* on client progress and strengths. On the other hand, unconditional positive regard (Raskin, 2001, p. 11331) is “not conditional or limited” (Merriam-Webster, 2023). Therefore, unconditional positive regard aligns well to the profession’s ethics principle to “treat others with compassion, dignity, and respect” (BCBA, 2020, p. 4) and reflects a social justice framework, acknowledging that “human life is to be universally cherished and valued” (Pritchett et al., 2022, p. 1088).

Building on this universal acceptance, Leland and Stockwell (2019) operationalized affirming care (for transgender and gender-non-confirming clients) within the behavior analytic literature as “access [to] valued reinforcers and resources at similar rates to those of [majority groups] . . . minimizing coercive contingencies . . . as well as [supporting] their authentic verbal behavior and expand networks of choice” (pp. 817–818). Extending to affirming care of Neurodivergent and disabled individuals, other professional disciplines have offered neurodiversity-affirming (Dallman et al., 2022), neurodiversity-affirmative (Shaw et al., 2022), neuro-affirming (Roux et al., 2023), neurodiversity-informed (Schuck et al., 2021), and neurodiversity-oriented (Hotez & Onaiwu, 2023) approaches within their scholarly literature. These professions include but are not limited to pediatrics (Hotez & Onaiwu, 2023), psychiatry (Shaw et al., 2022), psychotherapy (Chapman & Botha, 2023), occupational therapy (Dallman et al., 2022), speech-language pathology (Donaldson et al., 2017), and dentistry (Murphy et al., 2023). Chapman and Botha (2023) describe neurodivergence-informed therapy as that which “resists default normalization, is sensitive to neurodivergent perspectives, understands disablement as relational and political, and considers disability as a potential source of community and *pride*” (p. 315; emphasis added). Therefore, neurodiversity-affirming care requires not only prizing the person unconditionally or *noncontingently* but their disability identity. We hope to demonstrate that prizing the individual and characteristics of their disability is not incompatible with pursuing person-centered behavior change in areas that enhance quality of life. Affirming care is ethical care. Therefore, building on the *Ethics Code for Behavior Analysts* (2020), these co-authors offer that neurodiversity- or disability-affirming ABA may be defined as service delivery that (1) ensures the rights and dignity of Neurodivergent or disabled individuals (2) by actively pursuing and being responsive to all forms of Neurodivergent or disabled individuals’ self-advocacy including both vocal or nonvocal verbal behavior (3) to inform goal selection; selection and implementation of assessment, intervention, and progress monitoring methods; and interpretation of results in order to (4) promote behavior change that maximizes client choice, autonomy, self-determination, and quality of life. Through the calls to action and accompanying affirming actions, suggestions for how behavior analysts can engage in neurodiversity- or disability-affirming ABA are offered. Readers are encouraged to consider these affirming practices not only in the context of one’s professional practice, but also within “oppressive systems” (Pritchett et al., 2022, p. 1088).

## Calls to Action

### Call to Action 1: Identity and Language—“I shouldn’t need to remind anyone that I’m a person.”

#### Disability, Identity, and Intersectionality

Language evolves continuously and reflects the changing attitudes within and toward diverse communities and cultures (Andrews et al., 2019). Reclaiming words that may once have held a negative connotation for a historically underrepresented and under-resourced community is an example of this evolution. The viral hashtag #SayTheWord reflects the disability community’s efforts to reclaim the term “disability” in response to euphemisms such as “differently abled” or “special needs,” which unintentionally promote the belief that disability is shameful and should be avoided (Forber-Pratt, 2020, para. 2). In 2013, autistic disability rights advocate Lydia X. Z. Brown opined:

When I say that I am “disabled,” I am not putting myself down, insulting myself, suggesting that something is wrong with me, or making a negative statement about myself. I am staking a claim in an identity that is important to who I am as a person. I am recognizing that my mind/body function atypically. . . . I am not reducing myself to my disability, just as I am not reducing myself to my gender or race when I say that I am genderqueer or that I am Asian. Being disabled is one part, albeit an important part, of my multifaceted identity.

As Brown highlights, identity is complex and highly individualized. Much variability exists in preferences and experiences.

Intersectionality promotes an understanding of identity that reflects the interaction among categories such as gender, ethnicity, race, disability, social class, and sexual orientation (Crenshaw, 1989). This interaction results in an individual’s unique experience of oppression (Crenshaw, 1989). The experiences [or learning histories] of Autistic women; Autistic nonbinary individuals; and Autistic Black, Indigenous, or People of Color, for example, are distinct from the experience of white Autistic males. Liasidou (2013) highlights the additional barriers that a person with a disability will encounter if they have other marginalized identities. Onaiwu (2020) states, “the typical ‘face’ of autism tends to be that of a little white boy, regardless of autism’s actual prevalence in all racial, age, and gender groups” (p. 270). Stereotypes surrounding autism result in greater marginalization for oppressed groups and can be dire for Autistics who do not conform to expected norms. For example, police killed Elijah McCain, a Black Autistic individual, because they perceived his mannerisms as aggressive rather than associated with autism (Onaiwu, 2020).

Gender identity and sexuality are also more varied within the Autistic population than the non-Autistic population (e.g., Corbett et al., 2023; George & Stokes, 2018; Warrier et al., 2020). Approximately 11.4% of Autistic adults reported a desire to be the opposite gender from their gender assigned at birth, compared to 3% to 5% in the general population (van der Miesen et al., 2018). Transgender and gender diverse individuals were Autistic three to six times as often as cisgender individuals (Warrier et al., 2020). Nearly 70% of Autistic individuals reported being nonheterosexual compared to 30% of allistic (i.e., non-Autistic) individuals, with Autistic respondents reporting higher rates of homosexuality, bisexuality, and asexuality (George & Stokes, 2018). The implications for increased and different sexual education (Davies et al., 2022) and, as a result, the greater probability of negative mental health impacts (e.g., George & Stokes, 2018) merit provider awareness. These identities and intersections of identities are reflected in the way people refer to themselves and in the manner they wish others to refer to them.

#### Identity-First and Person-First Language

Jim Sinclair, an Autistic individual and an autism rights advocate, in his seminal *Don’t Mourn for Us* (1993) described his Autistic identity, “Autism is a way of being. . . . It is not possible to separate the autism from the person—and it if it were possible, the person you’d have left would not be the same person you started with” (p. 1). Identity-first language (IFL) aligns with Sinclair’s conceptualization and favors the use of “Autistic person" instead of “person with autism” (i.e., PFL). As the term suggests, identity-first terminology emphasizes autism as an identity. Brown (2011) notes that “Autistic individual” or “Autistic,” for example, indicates that autism may be one of a person’s core identities. IFL combats the connotation that “it is ‘bad’ to have a disability” (La Forge, 1991, p. 51) and asserts that Autistic and Neurodivergent people (or any disabled individual) should not have to remind anyone that they are a person.

Although PFL originated in the 1970s (Botha et al., 2021), it entered everyday use approximately 20 years later and continues to represent the standard in most training and professional circles, scholarly publications, and within legislation (e.g., People First Respectful Language Modernization Act of 2006). Use of PFL is so widespread that many individuals find themselves being corrected for the manner they talk about *themselves* (Kapitan, 2017). The rationale for the use of PFL at the time of its inception was to ensure an individual was not “defined by a diagnosis” and that the label, instead, became a “characteristic” (Collier, 2012, p. 1977). Many parents and caregivers of Autistic individuals, among others, have favored person-first language and been critical of the conceptualization of autism as an identity, noting the aspects of their child or loved one’s autism that prevent the individual from communicating or result in severe self-injury or aggression and that they believe are not reflected in the mainstream depictions offered by Autistic advocates (Singer, 2022).

In contrast to those with preferences for PFL, Andrews et al. (2013) suggests that PFL may have “overcorrected” to the extent of “further stigmatizing disability” (p. 237). This sentiment is echoed by others who suggest that this terminology may stigmatize terms that were “never considered derogatory or pejorative in the first place” (Collier, 2012, p. 1977). For those terms that may be considered derogatory, PFL has done little to reduce negative perceptions of stigmatized conditions (St. Louis, 1999). As evidenced across social media platforms and more recent scholarly works, the Autistic and Neurodivergent community’s views on the use of language suggest a trend toward group preference for IFL compared to PFL. Taboas et al. (2023) found that over 85% of Autistic adults (*n* = 298) and nearly 70% of parents/caregivers (*n* = 81) in the United States reported a preference for IFL terms. Yet, over 65% (*n* = 206) of professionals (e.g., teachers, therapists, clinicians) reported a preference for PFL terms (Taboas et al., 2023). Likewise, in a survey, 88.6% of the over 800 Autistic adults surveyed reported a preference for IFL (Organization for Autism Research [OAR], 2020). Despite the group preferences for IFL, the heterogeneity by members within this community is reflected in these surveys, with some Autistic respondents favoring PFL or other terms. In addition, these emerging group preferences likely overemphasize the voices of Autistic adults whose vocal verbal behavior is more readily understood by listeners. Although the preferences of minimally [vocal] verbal, nonspeaking, and younger Autistic individuals may be less apparent, their perspectives are also valuable and merit attention.

As a result of the greater awareness of shifting group preferences, many individuals and groups have incorporated IFL. For example, the *American Psychological Association Style Publication Manual, *Seventh Edition, now suggests that bias-free language includes PFL or IFL based on the individual’s or community’s preferences, placing expressed preferences before style, and encourages avoidance of “euphemisms that are condescending” (e.g., “special needs”; APA, 2022, negative and condescending section). This reflects important changes from earlier editions, particularly given the APA’s troubled history with the disability community (Andrews et al., 2019). Gernsbacher (2017) argues that IFL in scholarly writing is a disability rights, equality, and diversity issue. This evolution has meaningful implications for scholarly writing, as many journals defer to APA style guidelines. One Autistic individual in an OAR (2020) survey shared, “When a publication uses the word ‘autistic’ . . . I feel seen and accepted.” Yet, IFL has largely been absent from behavior analytic vernacular and scholarly writing.

Engaging in “person-*centered* language” (emphasis added) ensures that our verbal behavior “affirms agency and humanity” (Kapitan, 2017). To do so, the actions of the behavior analyst may involve asking or observing how people describe themselves and how they would like others to identify them, when applicable. Use of effective and socially valid approaches for enhancing the self-advocacy of clients while also increasing our commitment to pursue greater understanding of the complex perspectives of all our clients is essential. There is no one-size-fits-all approach and nuance is necessary; “I will . . . vary my language to suit my audience,” and use IFL with those who prefer it and vice versa (Cohen-Rottenberg, quoted in Collier, 2012, p. 1978).

#### Functioning Labels

Language that affirms diversity also requires abandonment of functioning labels (e.g., high functioning, low functioning; National Centre for Mental Health [NCMH], 2019). The term “high functioning” by medical providers at its origin was used to describe Autistic individuals without an intellectual disability (Alvares et al., 2020). This terminology has since broadened to widespread use beyond the medical community and expanded to include Autistic individuals who may have strengths in language, cognitive abilities, and adaptive skills (Alvares et al., 2020). Although widely used, Kat Williams asks us to consider whether such terminology promotes dignity, “Just think about how bizarre it would feel if Autistic people started asking you whether you were a high functioning non-Autistic person or a low functioning non-Autistic person. . .” (NCMH, 2019). Further, the overarching goal of terminology should be to convey client strengths and needs (Lei et al., 2021), the dichotomy of “high” versus “low” functioning fails to acknowledge the unique profiles of strengths and needs of clients and lacks the specificity needed to be useful in behavior analytic service provision. Individuals described as “high functioning” may be unable to access necessary supports or services. On the other hand, Autistic and Neurodivergent individuals globally labeled as “low functioning” often fail to have their strengths acknowledged and are restricted from accessing certain programs, services, or activities. Functioning labels ignore the heterogeneity of strengths, needs, and supports within individuals of all neurotypes and the changes within these profiles over time. For example, Mary Doherty (2023), who is an Autistic woman, notes that although she is a high-achieving medical professional, there are challenging times when she is unrecognizable; “I have no access to fluent speech . . . a complete lack of interaction . . . favored comfort items, intense stimming and time is what it will take for me to regulate to the point at which I can once again communicate using spoken words” (para. 2). Instead, the systematic assessment of the strengths and needs of clients, a cornerstone of behavior analysis (BACB, 2017), provides the specificity necessary to inform effective behavior analytic interventions, rendering ableist and ambiguous “functioning” labels to be *non-*functional.

#### Neurodiversity-Affirming Actions: Identity and Language

Our ability to effect meaningful changes in behavior that enhance the quality of life for those we serve requires “effective communication [which] depends on choice of language” (Carr, 1996, p. 625). Aiming to be respectful to the [identities and] preferences of individuals/groups (Reid et al., 2018) instead of demonstrating allegiance to professional traditions (Bottema-Beutel et al., 2021) provides the rationale for adapting how we use language with and regarding those whom we support. Reid et al. (2018) models the self-reflection necessary to adapt personal practice, noting that had he been responsive to the stated preferences of self-advocates and their families, “his naming practices likely would have changed and been perceived as more dignified at a much earlier time” (p. 73). Not only what we do, but also what we say, conveys meaning regarding a person’s worth or “importance” (Council on Quality & Leadership, 2017, p. 35). Our words reflect our values and ethics as behavior analysts. For individuals who are not Autistic or Neurodivergent, advocacy should reflect allyship; critical to the conceptualization of ally is the emphasis on action. Identifying and making conscious choices about language is an actionable way to demonstrate allyship with the Autistic and Neurodivergent communities. After all, we “should select words for their impact on the listener, not on the speaker” (Skinner, 1957, as cited by Lindsley, 1991, p. 449). Bottema-Beutel et al. (2021) provides questions to guide self-reflection to prevent ableist language, such as whether the individual would use the language in conversation with an Autistic person, if the language perpetuates beliefs that Autistic individuals need to be “fixed,” and whether the language chosen was done so exclusively due to professional traditions as opposed to the preferences of Autistic individuals (p. 25). Table 1 summarizes suggestions for language and communication with and about Autistic and Neurodivergent individuals.

**Table 1:** Neurodiversity-affirming actions: Concerns and suggestions for language and communication

Concerns	Suggestions for Change
Speaking about clients in front of them	Speak with clients (Reid et al., 2018)
Valuing only vocal forms of verbal behavior	Valuing and being responsive to all verbal behavior
Use of functioning labels (e.g., “high functioning,” “low functioning”)	Specific strengths, needs, and supports needed and/or wanted (Bottema-Beutel et al., 2021; Reid et al., 2018)
Use of term “deficit”	Use of term “difference”
Use of term “challenging behavior” or “problem behavior	Specific description of behavior (Bottema-Beutel et al., 2021), contextually inappropriate behavior, behavior that interferes with learning, unsafe behavior
Approaches involving “doing *to* clients” or “doing *for* clients”	Approaches involving “doing *with* clients” (National Center on Deafblindness, 2024)
Use of term “nonverbal”	Use of term “nonspeaker” (Autism Society, 2023), “minimally verbal,” or “nonvocal”
Deficit-focused approaches	Strengths-based approaches (Steiner, 2011)
Technical jargon	Communicate in a manner the listener understands (Becirevic et al., 2016; Rolider et al., 1998) so the consumer can make informed decisions (Autism Society, 2023)

Neurodiversity-affirming actions regarding the use of IFL and PFL are offered in Table 2.

**Table 2:** Neurodiversity-affirming actions: Context and suggestions for use of identity- and person-first language

Context	Suggestions for Change
Client preference is known	Match it (e.g., “follow the Autistic individual’s lead,” when a preference is demonstrated; Lei et al., 2021, p. 1349)
Client preference not known	Ask question(s) and observe to seek understanding (National Center on Disability & Journalism, 2021; Taboas et al., 2023)
Client and caregiver preferences are not aligned	Tailor to the individual present and pair terms when parties together (Collier, 2012)
Speaking to group	Match group preferences (if known) or use identify-first language for Autistic/Neurodivergent groups (Collier, 2012)
Dissemination to field of ABA	Behavior analytic researchers and behavior analytic publications should expand use and acceptance of identity-first language when preferred by individual/group referenced

### Call to Action 2: Dignity, Self-Determination, Choice, & Assent—“Eat too many donuts and take a nap” (Bannerman et al., 1990)

#### Dignity

Dignity, from the Latin word *dignitus* (merit) and *dignus* (worth; Kennedy, 2016), involves people being treated as worthy, valued, and respected (Reid et al., 2018). The *Ethics Code for Behavior Analysts* (2020), like Reid et al. (2018), employs a definition of dignity that aligns to the widely understood meaning by non-behavior analysts. For example, treating “others with compassion, dignity, and respect” is operationalized as ensuring equitable treatment regardless of differences, honoring privacy and confidentiality, promoting self-determination, and offering necessary information to allow clients to make informed choices regarding services (BACB, 2020, p. 4). Dignity is central to the conversation of human rights for all individuals, but particularly for individuals with disabilities who, as a community, have historically and continue to experience dehumanization by society (e.g., CESS, Blenkush & O’Neil, 2020; genocide, Ford, 2014; euthanasia, Longmore & Goldberger, 2000; and forced sterilization, National Women’s Law Center, 2022). Individuals with disabilities comprise approximately 17% of the world’s population and represent the largest minority group (World Economic Forum, 2023). Several human rights declarations have enshrined the dignity and rights for all, specifically for disabled individuals (e.g., United Nations [UN], 1948; UN, 2006). The Autism Society (2024) echoes this commitment to the basic human rights of Autistic individuals to “be treated with dignity and respect for their individual autonomy in making their own choices and decisions as it relates to their basic needs” (bullet 1). 

Despite its values, the field of behavior analysis has had difficulty communicating its commitment to client dignity (Bailey, 1991; Carr, 1996). Becirevic et al. (2016) and Rolider et al. (1998) compared the use of behavior analytic terminology (i.e., jargon) to nontechnical (i.e., conversational or colloquial) terminology and across two studies. Findings highlighted the negative impact jargon had on layperson understanding and comfort. Likewise, Bailey (1991) reflected, "our critics feel that we are obsessed with controlling others and have no respect for their freedom or dignity. . . . Somehow we neglected to develop socially acceptable terminology for presenting our concepts to consumers” (p. 447). Behavior analysts *can* create environmental conditions for clients that promote their dignity. Aligned to the profession’s ethical principles (BACB, 2020), these co-authors will offer behaviorally oriented solutions to enhance dignity by promoting client choice and self-determination as well as through client assent (or consent).

#### Choice and Self-Determination

Choice can be conceptualized as the client’s allocation of responding across stimuli, which, over time, may indicate relative preferences (Fisher & Mazur, 1997). Choice is often categorized as a means of being self-determined, which involves individuals making decisions to demonstrate control over their lives (Baer, 1998; Schloss et al., 1993). Self-determination as a construct originated in the 1990s and was used in the context of individuals with intellectual disability (Wehmeyer, 2020). Self-determination, in addition to standing as a human right (McCorquodale, 1994), has been positively correlated to greater quality of life (Lachapelle et al., 2005) and is protective against trauma resulting from the individual being or perceiving themselves as “powerless” (Rajaraman et al., 2022, p. 49). In Howell et al. (2019), findings of a systematic review demonstrated that choice was often preferred (even when available stimuli were less preferred) and this universal preference for choice was demonstrated across participants of various ages and diagnoses. This preference for choice has been demonstrated among nonhuman animals as well (e.g., Catania, 1980). For a recent overview of the choice literature, refer to Rajaraman et al. (2022).

Efforts to promote self-determination, particularly for individuals with communication differences, have often been limited in practice (Reid et al., 2001). There has also been a failure to sufficiently embed choice in behavior change procedures and to teach choice-making (Ferguson et al., 2019). For example, only 6% (i.e., 8 studies) of the 141 studies that addressed social validity included a choice of intervention (Ferguson et al., 2019). In addition, when choice opportunities have been embedded, these have been limited to only “minor daily decisions” (i.e., meal or food choices; Faw et al., 1996, p. 174).

Explanations for why behavior analysts have difficulty addressing self-determination and choice may include environmental or logistical barriers, the prioritization of client progress, and the biases related to the ability of disabled individuals to make decisions regarding their lives (Vaucher et al., 2020). Some logistical reasons highlighted within the context of adults with disabilities in a residential setting included an insufficient number of direct care staff, turnover on care management teams, lack of resources (i.e., time, space) for meetings between clients and staff, and resistance to change or the time required to advance new initiatives (Vaucher et al., 2020). These variables highlight the resources necessary in addition to the education and “vision” required to engage in practices that shift control from staff to clients.

Even in environments where the necessary resources exist, the perceived role of the behavior analyst to maximize client learning and skill development may impede the prioritization of choice. As Bannerman et al. (1990) aptly suggested, a balance must be struck between “the right to habilitation with the right to personal liberties” (p. 79). This also reflects a misalignment, between the desire for maximal independence *for* the client by the provider and the client’s acceptance of supports that may be necessary to enhance quality of life. This tension between the client’s and the provider’s priorities highlights the distinction made by many Autistic advocates regarding the preference for the goal of “autonomy” instead of “independence.” These advocates report that independence is likely an impossible goal as it requires or indicates the absence of support/support by others (Autistictic, 2018) whereas autonomy is currently positioned by the same groups as a better goal because “Autonomy can often be achieved in cases where independence can’t” (Autistictic, 2018, autonomy section).

Another critical variable that likely contributes to providers failing to promote opportunities for self-determination and choice reflects the provider’s belief regarding whether the client can make “responsible” choices (e.g., Baer, 1998; Bannerman et al., 1990). ASAN (2024b) has reflected on this denial of rights, “often, if anyone thinks the person with a disability cannot make good choices, the person with a disability has their right to make choices taken away” (introduction section). Yet, individuals prefer choice, and the benefits vastly outnumber the potential negative consequences (Bannerman et al., 1990). Even when harm may result, choice remains a cherished freedom (Blatt, 1987, as cited in Schloss et al., 1993, p. 216), and to prevent choice due to potential risks would impede dignity (Perske, 1972). Neither risk nor decision-making support are dichotomies; instead, various degrees exist (Schloss et al., 1993) that allow for maximization of client dignity, choice, and self-determination. To effectively promote client dignity and self-determination via choice-making opportunities and empowerment, choices must be frequent and meaningful (Brown et al., 1993). Brown et al. (1993) suggest that to do so, there must be a diversity of choice. For example, when a client is only offered the choice of a snack item, that individual is denied various dimensions of choice (e.g., *within* activities choice [wanted ice cream], choice to *refuse* [did not want a snack], choice of *with whom* [wanted to have snack alone]; Brown et al., 1993, p. 320). On the other hand, Faw et al. (1996) trained individuals to make decisions about their community living settings through skills training (e.g., identify preferences, ask questions and seek clarification, and evaluate options). This demonstration highlights the relationship between diversity of choice and the ability to demonstrate *genuine “*control” within one’s life (i.e., self-determination; Reid et al., 2001, p. 341). Finally, choice-making skills are necessary to agree to participate or assent in services (Breaux & Smith, 2023).

#### Assent

As many behavior analysts provide services to individuals aged 18 and under, informed consent by the parent or guardian is commonly the focus of behavior analytic ethical guidelines. However, whereas consent is required and legally binding, assent is not and is often sought only at the discretion of the provider (i.e., “obtaining assent from clients *when applicable*”; BACB, 2020, p. 11; emphasis added). Assent within a clinical content may be conceptualized as the uncoerced indication of willingness or approval to engage in an activity, such as assessment or intervention (Breaux & Smith, 2023). Assent withdrawal or dissent, on the other hand, reflects the individual’s unwillingness to engage in the assessment or intervention (Breaux & Smith, 2023). Breaux and Smith (2023) proposed “assent-based intervention” as services that prioritize and tailor assent and assent withdrawal procedures to the individual. Given these conceptualizations, we suggest that assent is *always* “applicable” if we are to engage in ethical, compassionate, and neurodiversity-affirming behavior analysis.

Behavior analytic literature regarding assent withdrawal procedures is limited (Breaux & Smith, 2023; Morris et al., 2021). In their evaluation of 187 behavior analytic empirical studies that included assent procedures, Morris et al. (2021) found that 28 articles, or 15% included “detailed” descriptions of assent procedures used. The categories of assent procedures used included the researchers (1) providing the participants a written, pictorial, and/or verbal description of the study; (2) asking the participants if they wanted to participate; (3) directly observing for behaviors aligned to participant assent or dissent; (4) requiring a psychomotor response (e.g., complete a smiley or sad face to indicate assent or dissent, respectively); (5) providing exposure to the procedures via a tutorial or practicing a sample of the procedures; and (6) using attention to increase the probability of assent (Morris et al., 2021). However, in the prior list, providing exposure to procedures before assent is obtained or attempting to increase the likelihood of assent may be considered coercive and thus may not functionally reflect assent. Autistic people value therapeutic approaches that emphasize consent (or assent) and perceive ABA as failing to do so (Gardner, 2017). These failures are evidenced in the themes reflected in the statements by Autistic adults who experienced ABA, who reported being unclear *that* they were in therapy and *why* they were in therapy, lack of clarity regarding the purpose of ABA or the activities they engaged in during services, and not finding the targets for behavior change to be meaningful (Anderson, 2022). This feedback may remind us that, along with the consent process, the assent process should also be *informed,* in order to enable our clients to serve as informed consumers of ABA.

#### Neurodiversity-Affirming Actions: Dignity, Self-Determination, Choice, and Assent

To effectively honor the rights of our clients and the individuals served through our science, prioritization of their dignity is paramount. Self-determination, choice, and assent are the means through which dignity can be realized within behavior analytic services. Regardless of the age, preferences, communication methods, strengths, areas for further growth, and preferred supports, using the appropriate methods to promote client self-determination and choice serves to prioritize dignity. Our ability as behavior analysts to develop repertoires that foster meaningful choice-making and self-determination is more limited, “by our lack of vision as to what is possible. . . . The only person who should be able to decide what they can and cannot do is the person themself" (Gerhardt et al., 2022a, p. 457).

Consider the affirming actions outlined in Table 3 for promoting self-determination and choice. These affirming actions align to the skill-building necessary and the collaboration required for clients to serve as and be treated as informed consumers.

**Table 3:** Neurodiversity-affirming actions: Guidelines for self-determination and choice

•Ensure dignity by aligning services to the client’s stated/demonstrated/known wants, needs, values, and preferences. When these are not fully understood, (1) be diligent in gaining understanding; (2) seek to be responsive to demonstrations of momentary assent and assent withdrawal; and (3) engage in self-reflection to inform how one may want their own loved one treated (Reid et al., 2018).
• Seek client’s input and feedback in an ongoing manner regarding (1) the goals of intervention; (2) the procedures (i.e., assessment, intervention); and (3) outcomes or effects, intended or unintended (Wolf, 1978).
• Embed choice opportunities as often as possible.
• Balance client benefit and risk (Bannerman et al., 1990; Perske, 1972). Where level of risk is unacceptable, mitigate via supports, not by removal of choice.
• Ensure that choice opportunities do not solely originate from the provider (Brown et al., 1993) and that staff are responsive to demonstrations of choice or preference by clients outside of programmed opportunities.
• Systematically teach and increase choice-making repertoires, affording meaningful, increasingly complex, diversity of choices (Brown et al., 1993).

Foundational to client choice is the individual’s ability to determine whether to engage in services and research via assent or consent. To affirm the client or participant’s *human right* (even if not in a legal context) to opt in or out of services/research, consider the affirming actions outlined in Table 4.

**Table 4:** Neurodiversity affirming actions: Guidelines for client assent

• Assent should be sought even when it may not be *required* or when waived.
• Employ an *informed* assent process.
• Seek assent in the manner that aligns to the client’s means of expressing agreement or disagreement. The Ethics Code (BACB, 2020) defines assent as “vocal or nonvocal behavior” that indicates the participant’s agreement to participate in research or behavioral services (p. 7).
• Consider other indicators of assent (e.g., approach behaviors with space, provider, instructional materials) or dissent/assent withdrawal (e.g., saying “no”/”stop,” requiring force to enter the area with the provider, attempting to elope from work area) (Breaux, 2021).
• Honor assent and assent withdrawal for the client over the course of assessment and intervention, even within the same session.
• Ensure opportunities to assent/withdraw assent are offered and/or responded to prior to experiences where an opportunity for harm exists (e.g., physical prompting; Breaux & Smith, 2023).
• Develop and train those directly working with the individual on the operational definitions of assent, dissent, and assent withdrawal for a given client (Breaux & Smith, 2023), with the acknowledgment that different activities may allow for different indications of assent, and train staff how to proceed if dissent or assent withdrawal occurs, including session termination criterion (Breaux & Smith, 2023).
• Measure opportunities for assent/assent withdrawal and occurrences of assent and assent withdrawal (Breaux, 2021; Breaux & Smith, 2023). Use these data to inform adaptations to environmental conditions (e.g., goals, assessment procedures, intervention procedures, provider/staff, timing) that may serve to evoke assent when subsequently sought.

### Call to Action 3: Social Validity- “Who decides what is socially significant?”

The *applied* dimension of ABA emphasizes that the science is utilized to address behaviors of socially meaningful impact (Baer et al., 1968, 1987). Wolf (1978) further operationalized social importance by outlining three dimensions of social validity: (1) goals; (2) procedures; and (3) the (intentional and unintentional) effects of services.

#### Participatory or Community-Informed Approaches

Research in ABA, which informed our evidence-based practices, has largely overlooked the importance of participatory approaches (Pritchett et al., 2022) or methods that render community members partners in the decision-making process (Nicolaidis et al., 2019). Pritchett et al. (2022) highlight that in failing to reflect the input of those with whom we support, a disparity is created, which perpetuates “power imbalances” (p. 1088) that interfere with our pursuit of social validity. Fawcett discussed the alignment of the values of ABA to “community research and action” (p. 622) in a 1991 issue of the *Journal of Applied Behavior Analysis*. Through shared decision making between researchers and participants, studies may be designed, their results interpreted, and their findings disseminated (Fawcett, 1991). Therefore, community-based research is not only reflective of social validity (Wolf, 1978; Schwartz & Baer, 1991) but also to the dimensions of ABA (e.g., applied; Baer et al., 1968, 1987). There is now a strong impetus for behavior analysts to ensure collaboration at all levels with those we are serving, and to understand their lived experiences and preferences (Miller et al., 2019). In doing so, participatory methods may allow behavior analysis to contribute to efforts to “understand and change the world” (Fawcett, 1991, p. 634) in pursuit of social justice, particularly for and *with* “those whose voices have been omitted” historically (Pritchett, 2022, p. 1074).

#### Identifying and Centering the Client

Although behavior analysts have lauded the field’s emphasis on social validity, too often the processes to evaluate social validity and determine social significance have excluded the client in favor of proxy-reporting (e.g., parent/caregiver, teacher; Anderson et al., 2021). In a review of the literature, young children were rarely (i.e., fewer than 3% of cases) included in social validity assessments of the services they were provided (Heal & Hanley, 2008, as cited in Hanley, 2010). Yet, critical to the conversation of social significance is accurately determining who the client is at any given time or which individual(s) or organization is the recipient of the behavior analyst’s services (BACB, 2020). Behavior analysts in home settings are typically hired by the parent, and in consultation settings, they may be brought in by a school administrator, teacher, or related service provider. The presence of multiple relevant parties likely complicates the prioritization of the client’s best interest at times. For example, prioritizing the needs and interests of other relevant parties over that of the client has been a concern among neurodiversity advocates; Lynch (2019) asks, “Did it really help the child, though? Or the parents?” Brechin (2019) recommends determining and communicating who the client is, as well as the hierarchy of other relevant parties, at the outset of and throughout the service relationship.

The client’s age or the features of their disability (e.g., communication or cognitive differences) may limit the feedback they can provide regarding the social validity of the behavioral targets, procedures, and outcomes (Johnson, 2022). The historical reliance on social validity measures that require clients to verbally respond to provided questions often exclude clients with language challenges (Hanley, 2010). In these cases, it is increasingly important for the indirect consumers (e.g., caregivers) and the behavior analyst to remain cognizant of the power differential that exists. Criticisms of ABA reflect the profession’s failure to respect variations in human behavior that are central to Neurodivergent identity; as a result, the dignity of individuals is not protected and unintentional harm results (Shyman, 2016). On the other hand, neurodiversity-affirming behavior analysis can provide Autistic individuals supports that respect individual differences while addressing challenges and teaching life skills that promote autonomy (Johnson, 2022).

#### Assessment

Before addressing the selection of socially significant behavior, the role of assessment should be considered. Criterion-referenced assessments are a popular measurement tool utilized by behavior analysts, yet there are many aspects within their design and in the interpretation of the results produced that may reflect neuromajority bias. These assessments measure the proficiency of a skill by comparing the behavior to a mastery criterion and results are used to inform and evaluate interventions. Despite their merits, criterion-referenced assessments often utilize neuromajority development as a reference for the behaviors measured and the proficiency criteria (Dixon, 2015; Partington, 2010; Partington & Meuller, 2012; Sundberg, 2008). For example, the Verbal Behavior Milestones Assessment and Placement Program (VB-MAPP; Sundberg, 2008) is based on social and linguistic developmental milestones from birth to age 4. For example, if the behavior of an Autistic client does not meet the referential criterion, it may be identified as a deficit and interpreted as needing intervention. However, discretion is needed to discern whether the client’s performance in each assessment area reflects a *difference* or a deficit in need of intervention. When an excess or deficit in a behavior level compared to the assessment criterion could interfere with learning or cause potential harm, addressing the behavior would be socially significant. On the other hand, addressing variations in behavior that do not negatively affect learning or cause harm would lack social significance and could unintentionally harm the Autistic individual by attempting to “fix” an innocuous variation in behavior (Akhtar & Jaswal, 2020; Beck et al., 2020; Hull et al., 2021; Johnson, 2022). Johnson (2022) reflects, “Autistic people should not be held to neurotypical norms but rather those of their own community. Our behavior differences should not always automatically be pathologized” (p. 298). Assessment that includes a variety of sources and interpretation of findings that considers context is likely to enhance the selection of socially significant targets.

#### Selecting Socially Significant Goals

Behavior analysts are expected to consider the social significance of behavior change when selecting programming goals (BACB, 2017). However, this determination of significance should not rest solely with the behavior analyst. Use of person-centered planning offers a collaborative model through which goals meaningful to the client may be identified (Holburn, 2001; Smith et al., 2006). However, when caregivers or other relevant parties play an outsized role compared to the client (due to the client’s age or the impacts of their disability), the behavior analyst should consider *who* is benefitting from a change in behavior when selecting goals and writing behavioral change objectives. This is particularly relevant when the person whose behavior is being changed may not be the person who requested behavior analytic services. It is also important to consider who benefits the *most* from any given behavior change program. The greatest benefit must be placed on the person receiving the services. For example, increasing in-seat behavior may be a selected behavioral target because the student could gain access to less restrictive learning environments. A practitioner utilizing the social model of disability perspective would consider whether the current level of in-seat behavior is functioning as a barrier to accessing a less restrictive environment because (1) the learning activities in that environment must be completed while seated or (2) whether a flexible seating arrangement also supports the learning activities. Perhaps a flexible seating arrangement could be used to a certain extent, but not for every learning activity. We may ask ourselves whether the behavior of the client or those in the environment should change, or both, and engage in allyship and advocacy on behalf of our clients accordingly.

#### Social Camouflaging and Stereotypy

Concerns exist regarding behavior goals focused on ameliorating Autistic traits when those traits do not pose any risk to health and safety or interfere with learning (Leadbitter et al., 2021). Social camouflaging refers to a class of behaviors that function to “pass” as neurotypical by hiding (i.e., masking) Autistic traits (Hull et al., 2019; Jorgenson et al., 2020; Lai et al., 2017). This behavior class encompasses a wide range of behaviors (e.g., posture, prosody, movement, proximity, eye contact, facial expressions, hygiene, lexicon, vocal and nonvocal verbal behavior) and measures of this behavior like the Camouflaging Autistic Traits-Questionnaire (CAT-Q; Hull et al., 2019) are being developed and validated. There are many examples of studies in the published literature on interventions that reduce Autistic traits (Schuck et al., 2021), suggesting a history of teaching social camouflaging within autism services. Reduction of stereotypy has been a particular area of concern within the neurodiversity community due to the purpose these behaviors may serve for communication or self-regulation (i.e., self-management behavior). Many of these concerns stem from the rise in mental health problems within the Autistic population and the suspected impact of social camouflaging on mental health (Beck et al., 2020; Cage et al., 2018; Cassidy et al., 2020; Hull et al., 2021). The neurodiversity movement calls for a focus on teaching adaptive functioning instead of neurotypical functioning (Kapp et al., 2013; Leadbitter et al., 2021). An example of this approach would be focusing instruction on reliable communication skills rather than teaching the speaker to make eye contact with a listener while communicating.

Reducing repetitive and self-stimulatory behaviors that are a common Autistic trait (e.g., “stims”) have also historically been an area of focus in the published literature (Schuck et al., 2021). Although some repetitive behaviors can result in physical harm, isolation, and loss of learning opportunities, others may be unharmful and possibly serve other adaptive functions. Autistic respondents report the self-regulatory role that stimming serves (Kapp et al., 2019). Other repetitive behaviors, like vocal parroting seen during early language learning, serve a developmental function in early childhood (McLaughlin & Fleury, 2020). In addition, trauma-informed care approaches within behavior analysis call for practitioners to consider that behavior may also function to adapt and cope with past traumatic experiences (Rajaraman et al., 2022). Therefore, considerations should be made regarding the possible functions of repetitive behaviors and whether reducing those behaviors would result in an unintentional loss of a developmental opportunity or self-management behavior related to trauma. It is important to note that Autistic individuals may contextually choose when they do or do not wish to emit camouflaging behaviors. However, the rising concerns about the effects of camouflaging behaviors on the well-being of Autistic individuals (Beck et al., 2020; Cage et al., 2018; Cassidy et al., 2020; Hull et al., 2021) merit behavior analyst consideration.

#### Compliance

Following directions serves as a behavioral cusp for many individuals given its role in the development in a variety of other, higher order, behaviors across a variety of domains (Bosch & Fuqua, 2001; Rosales-Ruiz & Baer, 1997). However, there is a distinction between the behavioral repertoire of following multistep directions and compliance. Increasing compliance with adult instructions is a behavioral goal often seen in instructional programming and published literature (Lipschultz & Wilder, 2017; Losinski et al., 2017; Radley & Dart, 2016). Instructional programming for teaching compliance often classifies any instance of not following an adult’s direction as an error or noncompliant behavior. When clients are taught that following all adult directions is the only correct form of behavior, they lose the opportunity to self-advocate or functionally communicate (e.g., for support, a break, an alternative) as well as to provide or withdraw assent. In this case, behaviors that indicate choice or lack of assent are classified as noncompliant and may even be “corrected,” thus requiring compliance or failing to honor assent withdrawal. Offering choice, including the option of not participating in behavioral services, when working with a client or student is one of the core commitments of trauma-informed care (Rajaraman et al., 2022).

Related to this, there are concerns about the impact of emphasizing compliance on sexual health and safety (Gerhardt et al., 2022b). Research has shown high rates of sexual abuse and victimization among the Autistic population (Brenner et al., 2018; Brown-Lavoie et al., 2014; Cazalis et al., 2022; Tomsa et al., 2021; Warrier & Baron Cohen, 2021; Weiss & Fardella, 2018). This risk for sexual abuse and victimization may be higher for Autistic individuals with intersecting identities (Pecora et al., 2019, 2020). Thus, Gerhardt et al. (2022b) suggest teaching functional noncompliance is a safety skill. Teaching functional noncompliance teaches a client to discriminate conditions when saying “no” in response to adult directions is adaptive. Functionally noncompliant responses serve as self-advocacy, a means to convey preferences, and an important safety skill (Kishbaugh et al., 2022).

#### Neurodiversity-Affirming Actions: Social Validity

As Cummings (2000) suggests, “Change done to you is debilitating; change done by you is exhilarating” (p. 11, as cited in Smith et al., 2006). Behavior analysts have the opportunity to engage and center clients within their care; we offer the following guidelines (see Table 5) for practitioners and other decision-makers to reflect upon and consider to promote socially valid behavior analytic practice.

**Table 5:** Neurodiversity-affirming actions: Guidelines for social validity

• Identify the client or determine who is the recipient of the ABA services.
• Prioritize the client’s perceptions of social significance (BACB, 2020). Consider use of person-centered planning approaches (Holburn, 2001; Smith et al., 2006) to maximize the client’s role in their services.
• Target behavior change that improves the client’s quality of life from the client’s perspective (vs. caregiver’s or society’s). Identify quality of life as an explicit goal of intervention (Parenti et al., 2019).
• Respect behavior differences among people. Consider whether behavior is a barrier to individual’s quality of life or a societal barrier.
• Consider the degree of behavior change that is socially significant. If it is determined that changing a behavior is a socially valid goal, determine the *degree* of change that is meaningful for the individual.
• Only address stimming or stereotypy in situations where the behavior causes harm to the client or others in the client’s shared environment. Prioritize antecedent modifications when stereotypy interferes with learning opportunities and accept differences in rates of instructional opportunities. This may also include finding replacement behaviors, especially if the stimming or stereotypy may function to adapt and cope with trauma (Rajaraman et al., 2022).
• Incorporate opportunities for clients to provide and withdraw assent during programming and be responsive to their demonstrations of assent and assent withdrawal.
• Teach functional noncompliance behaviors where the client is taught specific conditions where “no” to adult instructions is a crucial skill for their self-advocacy repertoire as well as their physical and sexual safety (e.g., simulations of abductions; Gerhardt et al., 2022b).

## Conclusion

The field of behavior analysis has increasingly acknowledged the need for culturally humble (Wright, 2019) and responsive behavior analytic services (BACB, 2020, 2022b; Fong et al., 2016; Miller et al., 2019). Neurodivergent individuals reflect a substantial proportion of those who receive ABA services (BACB, n.d.). Therefore, widening the conversations about the importance of diversity, equity, inclusion, and accessibility to include neurodiversity is critical in preventing unintentional harm (Johnson, 2022) that arises from a lack of understanding. However, the actions offered in this article are not (and cannot be) prescriptive, because it would be impossible to delineate rules applicable to all situations (Rosenberg & Schwartz, 2019). Ladson-Billings (2008) cautioned that approaches that instruct practitioners on specific actions for delivering culturally relevant pedagogy could lead to a one-size-fits-all approach that would not be responsive to individual learning histories and needs. Instead, committing to culturally humble and responsive pedagogy as a central principle can inform actions that are responsive to each client as an individual (Ladson-Billings, 2008; Wright, 2019) and which can affirm neurodiversity and disability.

We recognize that the calls to action in this article do not exhaustively address criticisms identified by the Autistic and Neurodivergent communities and we expect that additional calls may arise as the field continues to listen and reflect. The movement toward participatory approaches may support ABA to elevate consumers as partners (Fawcett, 1991; Pritchett et al., 2022). The voices of some within the Autistic community are not elevated within society due to differences in communication; yet, learning from those who “can describe their inner experiences” serves not to supplant the experiences of those within the community who cannot (Fletcher-Watson, 2022). Instead, this reflects the community regaining control of conversations from which they were excluded for decades (Fletcher-Watson, 2022).

The field of behavior analysis has entered a period of introspection. Carr (1996), nearly 30 years ago, reflected on our field’s bewilderment that society doesn’t “listen” to us instead of us asking ourselves “why have we not listened to society?,” noting, “We have much to offer. Nonetheless, until we make it clear that we too cherish society’s highest values, speak its language . . . we should expect to be ignored; and we will be” (p. 263). These calls to action ask behavior analysts to consider the role of societal ableism reflected within behavior analysis. Furthermore, the calls are critical of ableist practices and not the principles of behavior. It is essential to discriminate between criticisms as an issue of ethics rather than an issue of science. We can be responsive to poor social validity regarding ABA by engaging in meaningful and sustainable changes not only within our professional practice but also in the systems and structures in the field of ABA. Anti-ableist ABA including allyship and advocacy can contribute to detecting and dismantling policies and practices that fail to affirm the humanity and identities of Neurodivergent and disabled clients. Pritchett et al. (2022) suggests that, “We have to acknowledge the oppressive systems in our world and that these oppressive systems contribute to the inequities experienced by members of society with vulnerabilities. . . . Social justice is difficult labor . . . discomfort . . . is a symptom of change and progress” (p. 1089). Moving beyond performative allyship (Sylvain et al., 2022), using participatory approaches will include Autistic and Neurodivergent individuals within the field of ABA, its professional and corporate leadership, its research ranks and human subjects review boards, and in consultation capacities in order to center members of the Autistic and Neurodivergent communities in decision making that affects them and others within their communities.

## Data Availability

No original data were collected for this article and there were no human subjects, thus informed consent was not required.

## Footnotes

1 LLA is a white, cisgender female in the neuromajority; LSM is an allistic, white, Jewish, and cisgender female; NS is an allistic, Brown, Muslim, and cisgender female; JFJ is a Black, Jewish, autistic, and cisgender female; and AB is a neurodivergent, Hispanic, cisgender male.

2 It is important to note that whereas the majority of certificants under the BACB reside in the United States, behaviors analysts practice throughout the world (BACB, n.d.). Therefore, although we cannot cover all countries within the scope of this article, we will briefly cover history in the United States.
